# Changes in retinal multilayer thickness and vascular network of patients with Alzheimer’s disease

**DOI:** 10.1186/s12938-021-00931-2

**Published:** 2021-10-03

**Authors:** Xi Mei, Conglong Qiu, Qi Zhou, Zhongming Chen, Yang Chen, Zemin Xu, Chenjun Zou

**Affiliations:** 1 Kangning Hospital of Ningbo, Ningbo Kangning Hospital, Zhuangyu South Road 1#, Ningbo, Zhejiang China; 2 Ningbo University, Ningbo, Zhejiang China

**Keywords:** Retinal nerve thickness, Retinal vascular network, Alzheimer’s disease, Biomarkers, Diagnostic method

## Abstract

**Background:**

Retinal biomarkers of Alzheimer’s disease (AD) have been extensively investigated in recent decades. Retinal nervous and vascular parameters can reflect brain conditions, and they can facilitate early diagnosis of AD.

**Objective:**

Our study aimed to evaluate the difference in retinal neuro-layer thickness and vascular parameters of patients with AD and healthy controls (HCs).

**Methods:**

Non-invasive optical coherence tomography angiography (OCTA) was used to determine the combined thickness of the retinal nerve fiber layer (RNFL) and ganglion cell layer (GCL), as well as the full retinal thickness (FRT). The vascular branching (VB), vascular curvature (VC), and vascular density (VD) for AD and HC groups were also obtained. The Mini-Mental State Examination (MMSE) was used to evaluate the cognitive performance of all the participants. After obtaining all the parameters, two-way analysis of variance (ANOVA) was used to compare the mean values of all the retinal parameters of the patients with AD and the HCs. Pearson's correlation was used to test the association between retinal parameters, MMSE scores, and vascular parameters.

**Results:**

Seventy-eight eyes from 39 participants (19 AD and 20 HC; male, 52.6% in AD and 45.0% in HC; mean [standard deviation] age of 73.79 [7.22] years in AD and 74.35 [6.07] years in HC) were included for the analysis. The average RNFL + GCL thickness (106.32 ± 7.34 μm), FRTs of the four quadrants (290.35 ± 13.05 μm of inferior quadrant, 294.68 ± 9.37 μm of superior quadrant, 302.97 ± 6.52 μm of nasal quadrant, 286.02 ± 13.74 μm of temporal quadrant), and retinal VD (0.0148 ± 0.003) of patients with AD, compared with the HCs, were significantly reduced (*p* < 0.05). Retinal thickness was significantly correlated with the MMSE scores (*p* < 0.05). Meanwhile, retinal VD was significantly correlated with the average RNFL + GCL thickness (*r*^2^ = 0.2146, *p* < 0.01). When the vascular parameters were considered, the sensitivity of the AD diagnosis was increased from 0.874 to 0.892.

**Conclusion:**

Our study suggested that the patients with AD, compared with age-matched HCs, had significantly reduced RNFL + GCL thickness and vascular density. These reductions correlated with the cognitive performance of the participants. By combining nerve and vessel parameters, the diagnosis of AD can be improved using OCTA technology.

*Trail registration* Name of the registry: Chinese Clinical Trail Registry, Trial registration number: ChiCTR2000035243, Date of registration: Aug. 5, 2020. URL of trial registry record: http://www.chictr.org.cn/index.aspx

## Background

In 2018, the National Institute of Aging and Alzheimer’s Association (NIA-AA) modified the diagnosis framework, suggesting that Alzheimer’s disease (AD) biomarkers, including β-amyloid (Aβ) deposition, pathologic tau (T), and neurodegenerative/neuronal injury biomarkers (N), can be added to the A/T/N classification system to improve diagnostic accuracy [1]. Furthermore, during the preclinical phase of AD, these biomarkers show abnormalities earlier than behavioral symptoms [2, 3]. However, we found that several biomarkers are assessed using expensive or invasive methods, including Aβ-positron emission tomography (PET), tau-PET, and lumbar puncture for Aβ or tau in cerebrospinal fluid. The retina and brain are connected directly by the axons of the optic nerve, which transport the amyloid precursor protein in retinal ganglion cells [4, 5]. Retinal and brain Aβ depositions are related. Therefore, retinal pathologies directly reflect brain pathologies.

One of the neurodegenerative biomarkers is the retinal structure, which can be evaluated noninvasively using optical coherence tomography (OCT) [6–8]. Retinal changes in AD patients are important for diagnosing and monitoring neurogenerative diseases [9]. In an OCT study, participants with AD showed that the ratio of the ellipsoid zone to the retinal pigment epithelium volume correlated with the cognitive assessment scores [10]. A quantitative histopathologic study revealed that the retinal nerve fiber layer (RNFL) was thinner in AD patients than in normal individuals [11]. The ganglion cell layer (GCL) thickness was also significantly reduced in patients with AD [12]. A 27-month pilot study showed a significant positive correlation between RNFL thickness and memory testing clinic questionnaire scores [13].

In addition to the decrease in retinal thickness in AD patients, there is an association between the degree of reduction in retinal thickness and the clinical severity of dementia [14–16]. In contrast, primary open angle glaucoma affects cognitive neurological functions besides vision; more visual field loss correlates with lower cognitive assessment scores [17].

The retinal vasculature is also important in AD patients [18, 19]. The participants with AD, compared with the patients with mild cognitive impairment (MCI) and controls, showed a significant reduction in retinal vascular density (VD, which was defined as the ratio of the number of pixels in the perfused retinal vascular area to the number of pixels of the entire retina), and changes in the vascular branches (VB, which was defined as the number of branches in the retinal central vascular system) and curvature (VC, which was defined as the average curvature of four main stem vessels in the retinal central vascular system) [20]. However, using only the retinal vasculature may not discriminate patients with AD from controls [21].

OCT has enabled us to visualize and assess the structures of the longitudinal retina and optic nerve, which is comparable to tissue slices [22]. En face OCT can reveal changes in structures parallel to the surface of the retina [23, 24]. OCT and en face OCT have been breakthroughs for observing portions of or the entire structures of the retina and optic nerve, and they have improved our perspective on the effects of diseases on the retina. Split-spectrum amplitude-decorrelation angiography was subsequently developed to observe retinal blood flow, and it has facilitated studies on the structure and function of the retina [25, 26]. This technology covers the conditions of the retina, optic nerve, and blood vessels to show the characteristics of the retina more comprehensively. The new generation of OCT angiography (OCTA) for clinical research has pioneering significance for the detection of neurodegenerative diseases.

In this study, we considered the coupling effect of blood vessels and nerves, and combined the vessel parameters to improve the diagnostic accuracy. We used OCTA to assess the retinal nerve and vasculature and comprehensively evaluate the diagnostic value of the fundus parameters. The combined thickness of the RNFL and GCL and the full retinal thickness (FRT) were used to evaluate the differences between the patients with AD and healthy controls (HCs). The correlations of retinal thickness with retinal vasculature were also investigated using Pearson’s correlation tests. In addition to neurological characteristics, vascular parameters can indicate neurological dysfunction. More objective assessments, apart from neuropsychological scales and especially the noninvasive modalities, are needed to increase the specificity and sensitivity of AD diagnosis.

## Results

### Demographic and cognitive characteristics of participants

After obtaining informed consent, 39 participants, including 19 patients with AD and 20 HCs, were recruited for this study. The demographics and clinical characteristics of the participants are detailed in Table 1. The mean age of the AD patients was 73.79 ± 7.22 years, 52.6% were male, body mass index (BMI) was 20.94 ± 2.34, duration of education was 9.00 ± 2.67 years, MMSE score was 12.79 ± 5.44 and mean disease course was 5.05 ± 3.17 years. There were significant differences in the mean MMSE scores between the AD and HC groups (*p* < 0.01). There were no differences in age, BMI, or level of education between the groups (*p* > 0.05).

**Table 1:** Demographic and clinical characteristics of all participants

Variables	AD patients (***n*** = 19)	Health controls (***n*** = 20)	p value
Age (year)	73.79 ± 7.22	74.35 ± 6.07	0.794
Sex (male, %)	10 (52.6%)	9 (45.0%)	-
BMI	20.94 ± 2.34	20.94 ± 2.33	0.997
Education years	9.00 ± 2.67	8.90 ± 2.75	0.909
MMSE	12.79 ± 5.44	28.05 ± 1.61	< 0.001
MMSE_orientation	6.05 ± 2.68	9.99 ± 0.01	< 0.001
MMSE_registration	1.84 ± 0.83	3.00 ± 0.00	< 0.001
MMSE_attention_calculation	1.05 ± 0.78	3.90 ± 0.91	< 0.001
MMSE_recall	1.37 ± 0.68	2.90 ± 0.31	< 0.001
MMSE_language	2.16 ± 1.50	7.30 ± 0.73	< 0.001
MMSE_visual_space	0.37 ± 0.50	0.90 ± 0.31	< 0.001
years of AD	5.05 ± 3.17	–	–

*BMI* body mass index, *MMSE* Mini-Mental State Examination, *AD* Alzheimer’s disease

### Retinal neuro-layer thickness in patients with AD and HCs

**Fig. 1:**
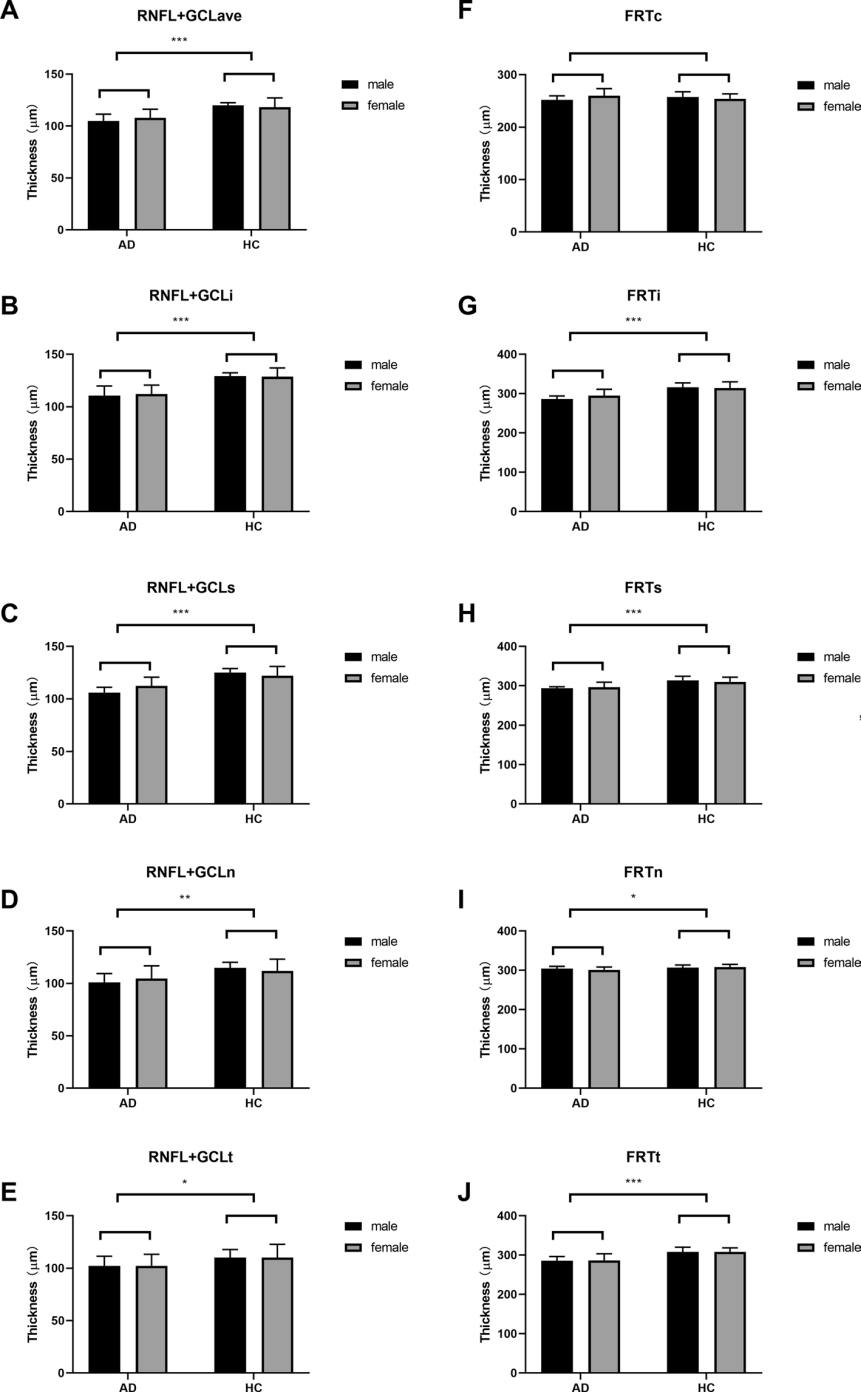
RNFL + GCL thickness (**A**–**E**) and FRT (**F**–**J**) in different regions in the AD and HC groups. The average RNFL + GCL thickness (**A**, *p* < 0.001), the RNFL + GCL thicknesses in the inferior (**B**, *p* < 0.001), superior (**C**, *p* < 0.001), nasal (**D**, *p* < 0.01) and temporal (**E**, *p* < 0.05) quadrants, and the FRTs of the inferior (**G**, *p* < 0.001), superior (**H**, *p* < 0.001), nasal (**I**, *p* < 0.05) and temporal (**J**, *p* < 0.001) quadrants were lower in the AD group than in the HCs. There was no significant difference between the FRT center thicknesses in the AD patients and the HCs (**F**, *p* > 0.05). * *p* < 0.05, ** *p* < 0.01, *** *p* < 0.001. AD, Alzheimer’s disease, HC, healthy control

The RNFL + GCL thickness and FRT of the two groups were obtained using OCTA. Figure 1 shows the comparison of the retinal thicknesses of the AD and HC groups using two-way ANOVA. The average RNFL + GCL thickness (106.32 ± 7.34 μm vs. 119.01 ± 6.71 μm, Fig. 1A, *F* (1, 35) = 30.87, *p* < 0.001) and RNFL + GCL thicknesses of the inferior (111.33 ± 8.67 μm vs. 128.97 ± 6.44 μm, Fig. 1B, *F* (1, 35) = 49.44, *p* < 0.001), superior (109.04 ± 7.35 μm vs. 123.44 ± 7.07 μm, Fig. 1C, *F* (1, 35) = 41.28, *p* < 0.001), nasal (102.83 ± 10.16 μm vs. 113.24 ± 9.11 μm, Fig. 1D, *F* (1, 35) = 11.21, *p* = 0.002), and temporal (102.26 ± 9.82 μm vs. 110.18 ± 10.39 μm, Fig. 1E, *F* (1, 35) = 5.61, *p* = 0.024) quadrants were significantly lower in the AD group than in the HC group. FRTs of the inferior (290.35 ± 13.05 μm vs. 315.11 ± 13.74 μm, Fig. 1G, *F* (1, 35) = 32.93, *p* < 0.001), superior (294.68 ± 9.37 μm vs. 311.57 ± 11.3 μm, Fig. 1H, *F* (1, 35) = 25.33, *p* < 0.001), nasal (302.97 ± 6.52 μm vs. 307.84.11 ± 6.51 μm, Fig. 1I, *F* (1, 35) = 5.39, *p* = 0.026) and temporal (286.02 ± 13.74 μm vs. 308.29 ± 10.72 μm, Fig. 1J, *F* (1, 35) = 30.03, *p* < 0.001) quadrants were also significantly lower in the AD patients. There was no significant difference between the FRT center thickness in the AD patients and the HCs (255.52 ± 9.77 μm vs. 255.72 ± 11.42 μm, Fig. 1F, *F* (1, 35) = 0.005, *p* = 0.946).

### Retinal vessel parameters of patients with AD and HCs

**Fig. 2:**
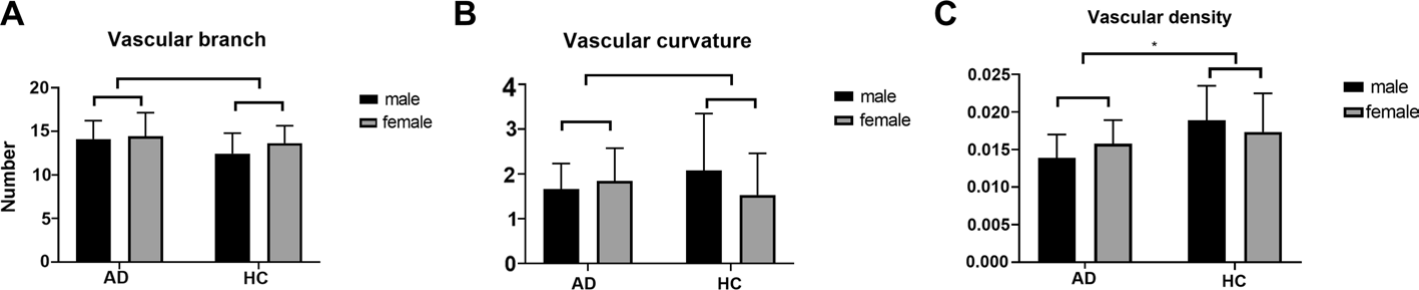
Vessel parameters, including vascular branch (**A**), vascular curvature (**B**), vascular density (**C**), of the AD patients and HCs. The vascular density was significantly lower in the AD group than in the HC group (*p* < 0.01). * *p* < 0.05

The vessel parameters, including VB, VC, and VD, of the AD patients and HCs were obtained (Fig. 2). There was a significant difference between the VDs of the AD and HC groups (0.0148 ± 0.003 vs. 0.018 ± 0.005, Fig. 2C, *F* (1, 35) = 6.054, *p* = 0.019). However, there were no differences between the VBs and VCs of the AD and HC groups (*p* > 0.05).

### Association of retinal thickness with MMSE

**Fig. 3:**
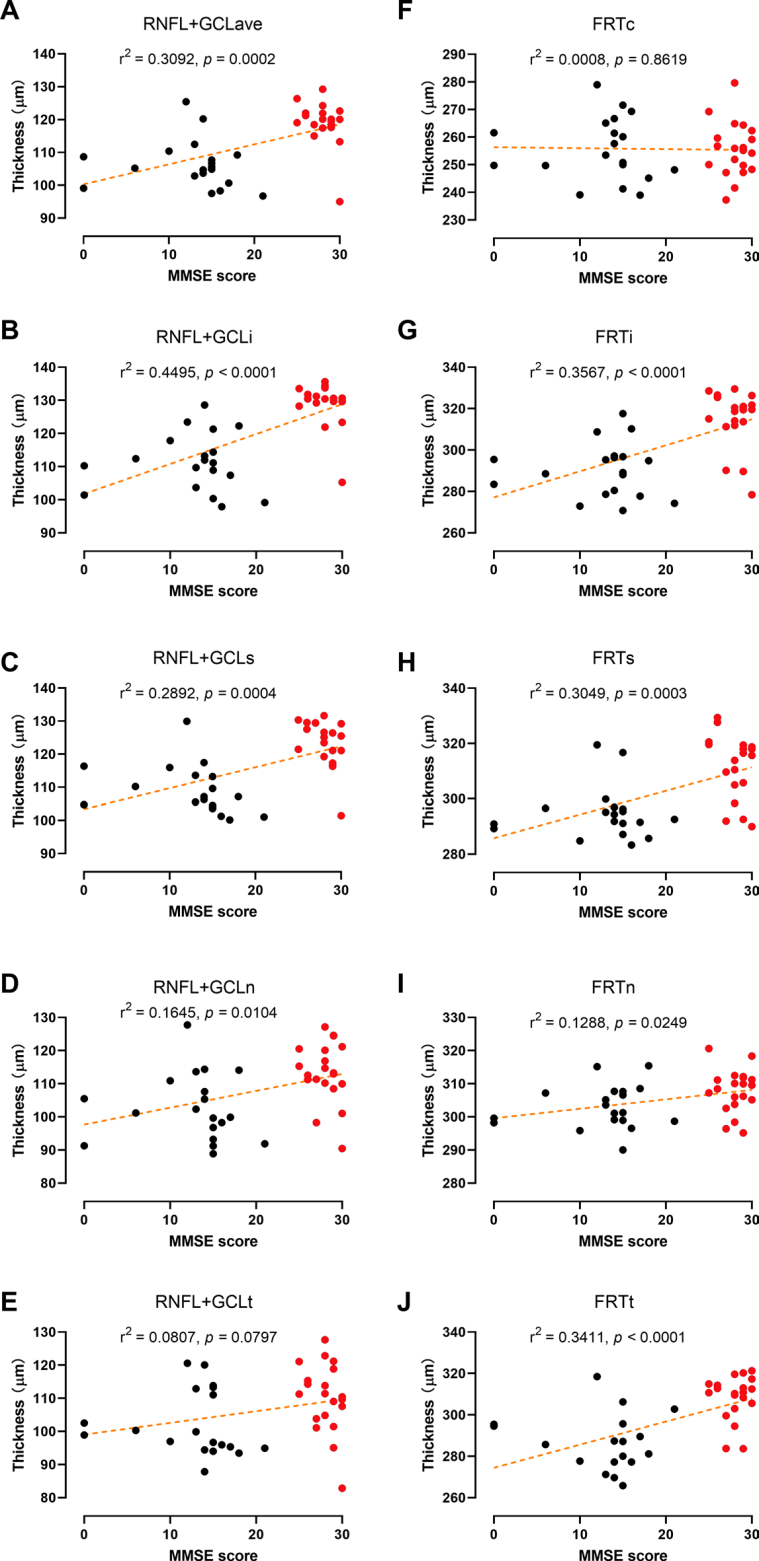
Sample correlations between regional retinal thickness parameters (**A**–**E**, RNFL + GCL; **F**–**J**, FRT) and MMSE scores of both AD patients and HCs. *RNFL* retinal nervous fiber layer, *GCL* ganglion cell layer, *FRT* full retina thickness

The correlations between the retinal thickness parameters and MMSE scores were determined and are shown in Fig. 3. The MMSE score were significantly correlated with the average of the RNFL + GCL thicknesses of all the five regions of interest (ROIs) (Fig. 3A, *p* < 0.001), and the RNFL + GCL thicknesses in the inferior (Fig. 3B, *p* < 0.001) and superior (Fig. 3C, *p* < 0.01) regions. The Pearson’s coefficients (*r*^2^) are presented in the figures. The MMSE scores was also correlated with the FRTs for the inferior (Fig. 3G, *p* < 0.001), superior (Fig. 3H, *p* < 0.01), and temporal (Fig. 3J, *p* < 0.01) regions but not for the central macular region (Fig. 3F, *p* > 0.05). For all the ROIs, retinal thickness was positively correlated with the MMSE score.

### Associations between retinal thickness and vascular parameters

**Fig. 4:**
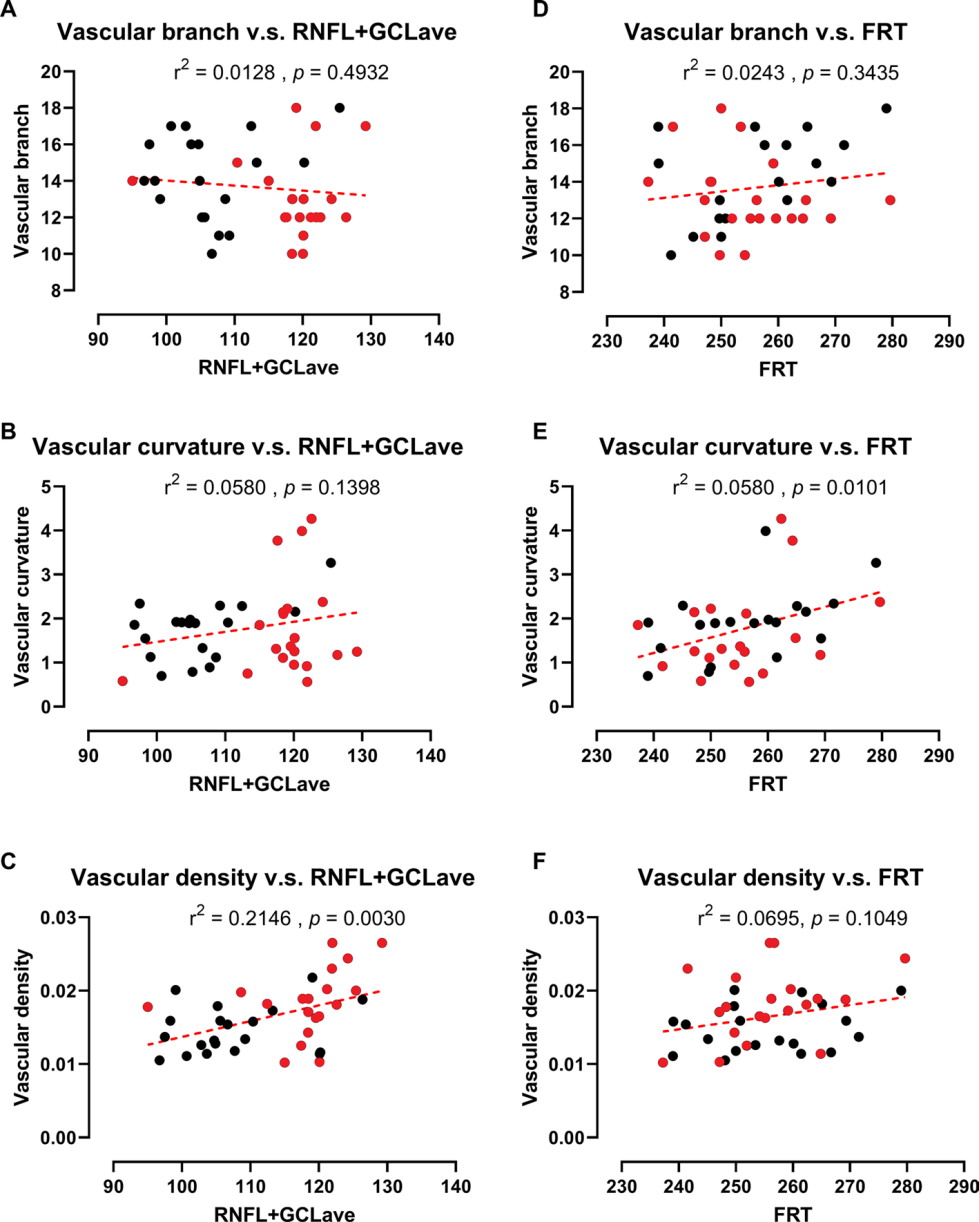
Sample correlations between variable retinal thickness and vascular parameters of both AD patients and HCs

For all the ROIs, Pearson's correlation was used to investigate the associations between retinal thickness and vascular parameters (Fig. 4). VB was negatively correlated with retinal thickness (Fig. 4A). VC and VD were positively correlated with retinal thickness (Fig. 4B–F). The average RNFL + GCL thickness was significantly correlated with VD (*r*^2^ = 0.2146, *p* < 0.01).

### Receiver operating characteristics (ROC) curve for RNFL + GCL, vessel parameters, and the combination of RNFL + GCL and vessel parameters

**Fig. 5:**
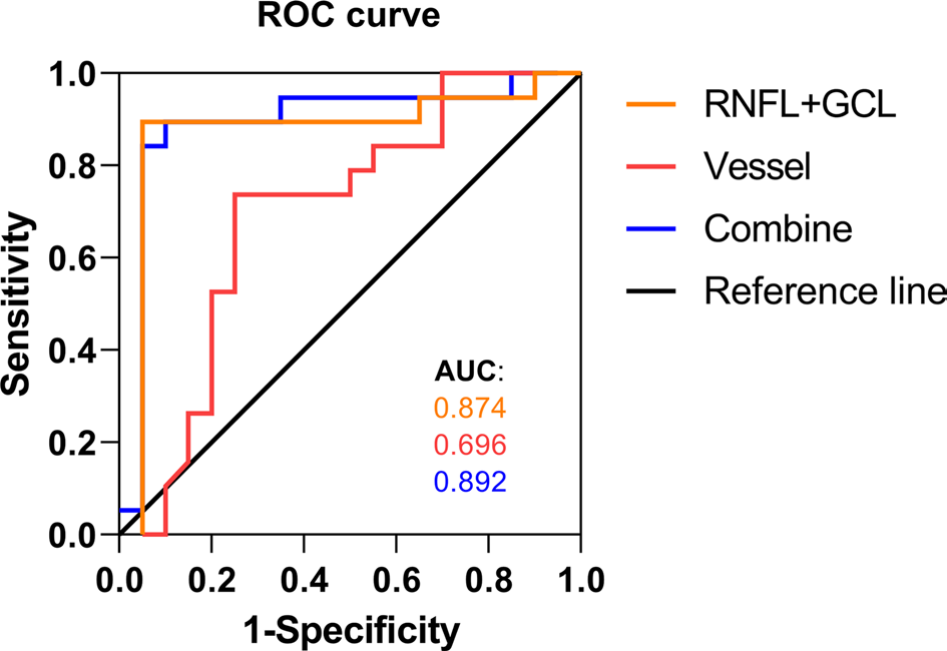
Receiver operating characteristics (ROC) curves for RNFL + GCL (orange line), vessel parameters (red line), and their combination (blue line). The areas under the curves were 0.874, 0.696 and 0.892, respectively

ROC curves show sensitivities (true-positive rate) and specificities (false-positive rate) of the RNFL + GCL (orange line), vessel parameters (red line), and their combination (blue line) for all participants with AD and HCs. The areas under the curves were 0.874 (95% CI, 0.7378 to 1.0000), 0.696 (95% CI, 0.5238 to 0.8683) and 0.892 (95% CI, 0.7705 to 1.0000) (Fig. 5).

## Discussion

Previous studies have used plasma biomarkers to assess the risk of conversion from MCI to AD, such as plasma P-tau181, Aβ_42_/Aβ_40_, and neurofilament light [27, 28], and they have often focused on the evaluation of individual biomarkers, rather than systematically determining the best set of “A/T/N” markers for individualized prediction. With technological advances, retina-related AD biomarkers can be incorporated into the diagnostic workup of AD. As confirmed by OCTA quantitative parameters, retinal neurodegenerations, involving the nervous system and vessels, may morphologically reflect and provide insights into brain conditions [29].

In this study, all quantitative retinal nerve parameters (RNFL + GCL thickness and FRT of superior, inferior, nasal, and temporal regions) were reduced in the eyes of AD patients relative to those of the HCs. The MMSE scores, which reflect cognitive performance, were lower in the AD patients than in the HCs. Besides the RNFL + GCL of the inner retina, the volumetric and thickness parameters of the outer retina and the Montreal cognitive assessment scores also decreased synchronously [10]. Although the patients in this study had moderate to severe AD, a significant decrease in the macular RNFL thickness was observed on OCT during the preclinical stage [30]. In clinical practice, the retinal thicknesses of AD patients and normal individuals may be comparable; the retina of normal people may be thin, and the conditions of these patients should be considered. OCTA can detect early alterations of the retina in patients with normal cognition [31]. The observation of retinal changes during the early stages of AD is beneficial for early diagnosis.

The retinal microvasculature was assessed using OCTA. Compared with the HCs, the patients with AD showed lower VDs. This can also be observed in patients with MCI [32, 33]. Furthermore, we investigated the relationship between the retinal microvasculature and thickness. There was a significant correlation between vascular density and RNFL + GCL thickness. These vascular changes may be caused by deposition of Aβ proteins [34]. Local microglial reactivity around Aβ plaques can affect retinal vein morphology and lead to a decrease in vascular density [35]. The retinal vascular and capillary networks can also provide valuable insights into the brain of AD patients [36, 37].

In addition to morphological changes, changes in the concentrations of various proteins and cytokines in the retina of patients with AD retina were present in mice with AD [38]. Moreover, non-invasive technologies are needed for the clinical diagnosis of AD, especially based on anatomical and functional changes [39]. Anatomically, the RNFL and GCL are parts of the third neuron of the inner retina; the RNFL is the axon, while the GCL is the cell body of the third neuron. Therefore, these two layers directly reflect the characteristics of the inner retina. Furthermore, the inner retina and brain share a common embryonic origin, which can directly reflect the characteristics of brain neurons. The OCT parameters of both global and specific retinal regions are needed to evaluate the accuracy of brain trajectory test [40, 41].

Several studies have proposed approaches to medical image procedding, including the elasticity-based state-space approach [42] and the Kalman filter snake method to improve the accuracy of diagnosis [43]. We used OCTA equipment (Cirrus 5000 Angioplex, Zeiss Meditec, Inc, Germany) with the integration of nerve and vessel segmentation methods, including optical microangiograph measurement [44].

This study did not involve patients with early-stage disease; however, the proposed non-invasive approach can be applied to early-stage disease. Patients are unwilling to undergo invasive testing during the early stages of the disease. Therefore, this study provides a non-invasive approach for early detection.

## Limitations

The limitation of this study is that (1) participants were relatively few. The follow-up study will involve a larger sample to improve the diagnostic accuracy. (2) The capillaries were not considered for the vascular density in this study. In further studies, capillaries will be included to allow a more accurate reflection of blood supply [45]. In the future, the severe stages of AD may be studied by considering the different courses of AD.

## Conclusion

To improve the accuracy of early diagnosis, biomarkers of AD should be evaluated as comprehensively as possible. In this study, a non-invasive optical method was used to assess retinal nerves and vessels; the conditions of retinal vessels and nerves may indicate conditions in the brain. Our study suggested that the AD patients, compared with age-matched HCs, had significantly reduced RNFL + GCL thickness and vascular density. These reductions correlated with the cognitive performance of the participants. In conclusion, retinal changes should be used as valuable clinical diagnostic information.

## Methods

### Participants

A total of 19 patients with AD and 20 age-matched HCs were recruited for this study. The AD patients with no retinal diseases aged between 60 and 88 years were diagnosed by two research psychiatrists according to the standards of the National Institution of Neurologic and Communicative Disorders and Stroke–Alzheimer’s Disease and Related Disorders Association (NINCDSADRDA) and the Diagnostic and Statistical Manual of Mental Disorders (DSM) [46, 47]. Medical evaluations, including neuroimaging and blood examinations, were performed to exclude alternative causes of dementia. The other inclusion and exclusion criteria, including Parkinson's disease, cerebral small vessel disease, chronic renal failure, cerebral small vessel diseases, white matter hyperintensities, and diabetes mellitus, were presented on the China website of clinical trials (clinical trial registration number: ChiCTR2000035243).

### Optical coherence tomography imaging

**Fig. 6:**
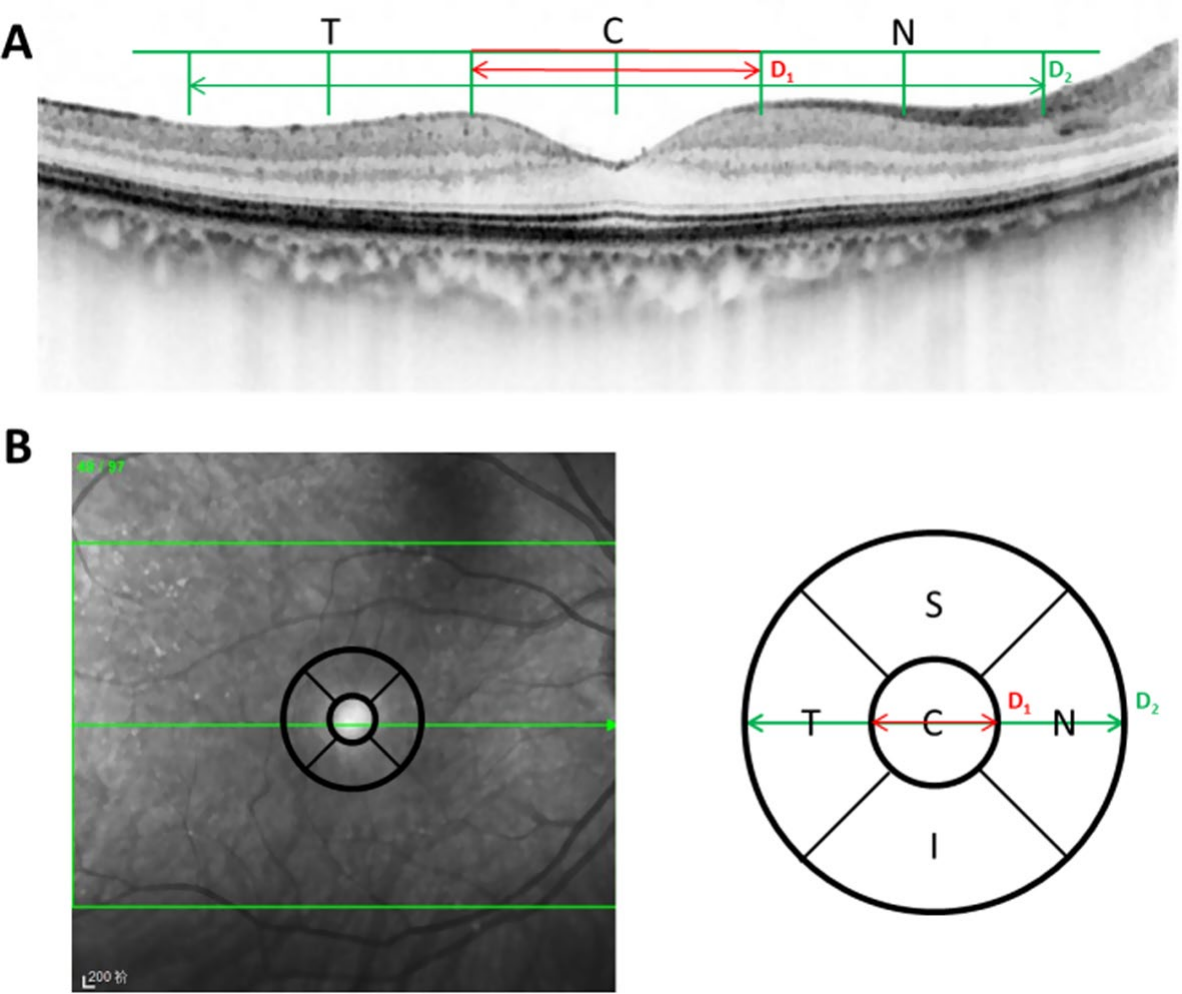
Segmentation of the retina using a B-scan (**A**) and en face projection (**B**). *S* superior sector, *T* temporal sector, *I* inferior sector, *N* nasal sector, *C* central sector, *D1* central diameter of macular, *D2* pFAZ diameter, *FAZ* foveal avascular zone. The segmentation (**A**) is labeled by the green line in **B**

OCT was used to measure the RNFL + GCL thickness and the FRT of the macular. Macular cube 512 × 128 scan patterns (Cirrus 5000 Angioplex, Zeiss Meditec, Inc., Germany) were used to obtain the parameters of the ROIs, including the peripheral macular region and center of macular region covering the foveal avascular zone (Fig. 6). The microvasculature in en face images were visualized. The VB (which was defined as the number of branches in the retinal central vascular system), VC (which was defined as the average curvature of four main stem vessel in the retinal central vascular system), and VD (defined as the ratio of the pixels covering the perfused retinal vessels to total pixels for the retina) for the area of the ROI were also measured. An image of each eye was obtained for each patient and each healthy participant. Seventy-eight images were obtained in total. The average values for the two eyes were obtained for all the parameters for each participant. The capillaries were not considered in the calculation of the vascular density in this study.

### Cognitive assessment

The MMSE scale, with scores range from 0 to 30, was used to evaluate the cognitive performance of all the participants. Lower MMSE scores indicated the poorer cognitive performance. The MMSE scale is currently the most widely used for cognitive examination, and it includes six dimensions: orientation ability, immediate recall, attention and calculation ability, delayed recall, language function, and visual space perception [48]. The scores were categorized as follows: ≥ 27 represented normal cognitive performance; 21–26 represented mild dementia; 10–20 represented moderate dementia; and < 10 represented severe dementia.

### Statistical methods

All data were analyzed using SPSS software V.16 (IBM Corp., Armonk, NY, USA) and are presented as the mean ± standard deviation. Differences in the data were assessed using two-way analysis of variance (ANOVA), followed by Bonferroni’s post hoc test (*p* < 0.05, with Bonferroni correction). An analysis of covariance (ANCOVA) was conducted to control for the effects of gender, age, disease duration, and education. The correlations between retinal parameters, MMSE score, and vascular parameters were tested using Pearson's correlation. A *p* value of < 0.05 denoted statistical significant.

## Data Availability

All data are included in the manuscript. However, the raw data used and/or analyzed in the present study are available from the corresponding author on reasonable request.

## Abbreviations

Aβ: β-Amyloid
AD: Alzheimer’s disease
ANOVA: Analysis of variance
BMI: Body mass index
FRT: Full retinal thickness
GCL: Ganglion cell layer
HCs: Healthy controls
MCI: Mild cognitive impairment
MMSE: Mini-mental state examination
OCT: Optical coherence tomography
OCTA: Optical coherence tomography angiography
RNFL: Retinal nerve fiber layer
ROC: Receiver operating characteristics
ROIs: Regions of interest
VB: Vascular branching
VC: Vascular curvature
VD: Vascular density
